# Patients' Attitudes Towards Integrating Environmental Sustainability Into Healthcare Decision‐Making: An Interview Study

**DOI:** 10.1111/hex.70155

**Published:** 2025-01-19

**Authors:** Eva S. Cohen, Dionne S. Kringos, Fleur Grandiek, Lisanne H. J. A. Kouwenberg, Nicolaas H. Sperna Weiland, Cristina Richie, Wouter J. K. Hehenkamp, Johanna W. M. Aarts

**Affiliations:** ^1^ Department of Obstetrics and Gynaecology Amsterdam UMC Location University of Amsterdam Amsterdam The Netherlands; ^2^ Amsterdam Public Health, Quality of Care Global Health Amsterdam The Netherlands; ^3^ Amsterdam Reproduction and Development Research Institute Amsterdam The Netherlands; ^4^ Centre for Sustainable Healthcare Amsterdam UMC Amsterdam The Netherlands; ^5^ Department of Public and Occupational Health Amsterdam UMC Location University of Amsterdam Amsterdam The Netherlands; ^6^ Department of Anaesthesiology Amsterdam UMC Location University of Amsterdam Amsterdam The Netherlands; ^7^ Department of Philosophy The University of Edinburgh Edinburgh UK

**Keywords:** climate change, environmental impact, environmental sustainability, green bioethics, green informed consent, shared decision‐making

## Abstract

**Introduction:**

The ethical obligation to reduce the environmental impact of healthcare systems prompts an exploration of if and when environmental concerns should be integrated into clinical decision‐making. In this study, we aimed to elucidate patients' attitudes regarding the provision of environmental information in healthcare decision‐making and to identify preferred approaches for integrating these considerations into patient–provider consultations.

**Methods:**

This interview study served as an in‐depth follow‐up of a survey study on gynaecological patients' perspectives on environmental sustainability within healthcare settings. We conducted semistructured interviews with 14 patients from two Dutch outpatient clinics between February and May 2024. We employed reflexive thematic analysis to analyse the data.

**Results:**

Five main themes were developed from the data: (1) Patients are an integral part of the transition to sustainable healthcare, (2) Patients are open to information on environmental impact of healthcare, (3) Information on environmental impact should be tailored to the individual patient and context, (4) Patients vary in preferences for involvement in decision‐making related to environmental sustainability and (5) Patients prioritize individual health over environmental concerns in healthcare decision‐making.

**Conclusion:**

The findings of our study underscore the importance of integrating sustainability into clinical decision‐making, aligning with bioethical principles and the expectations and goals of patients. By ensuring that environmental considerations are introduced in a personalized and context‐appropriate manner within patient–provider interactions, healthcare can foster greater support for sustainable practices.

**Patient or Public Contribution:**

Patients were involved in developing the pre‐interview questionnaire. The preliminary results of the study were presented to healthcare professionals from various backgrounds during a meeting of the Dutch Green Care Alliance, after which the input was incorporated into the interpretation of the study results. Finally, the complete manuscript was presented to representatives from the Dutch Patient Federation to obtain their input on the interpretation and implications of our research. The interpretation of our results aligned with the findings from their recent national inquiries, which included data from over 9300 patients within their network. Consequently, no changes were made to the manuscript's discussion.

## 1 Introduction

Transitioning to environmentally sustainable healthcare systems is globally recognized as imperative [1, 2, 3]. This acknowledgment of stakeholders, such as governments and healthcare professionals, is driven by the intricate relationship between healthcare delivery, environmental impact and health outcomes. It underscores the necessity for equitable and environmentally conscious healthcare practices to improve planetary health and wellbeing for all [4]. The rising focus on environmental sustainability in healthcare is reflected not only by the growing body of research on environmental impact of healthcare systems and potential mitigation strategies, but also through increasingly explicit policy ambitions [5, 6, 7, 8, 9, 10].

Patients could be important stakeholders in achieving ambitious sustainability goals within healthcare. Ultimately, the consultation room, the heart of our health system, is where some environmental trade‐offs might become tangible, shifting from policy ambitions to practical implementation that incorporates patient perspectives. Patients themselves are advocating for sustainability in healthcare practices. For example, a 2024 Dutch national survey among 9371 patients found that 69% of patients considered environmental sustainability an important aspect of healthcare delivery, and 73% expressed a willingness to incorporate sustainability factors into healthcare decisions [11]. A qualitative focus group study in the United States among patients and physicians found that patients encouraged climate and health counselling from physicians, especially when impacting their health [12]. Along these lines, multiple studies have suggested patient receptivity to ‘climate‐sensitive health counselling’, defined as the communication of the health effects of environmental hazards and the health benefits of climate mitigation strategies within healthcare encounters [13, 14, 15].

Despite studies showing patient support of sustainable healthcare systems, uncertainties persist regarding the integration of environmental considerations into healthcare decisions that may affect individual patient care, particularly in terms of ethics and shared decision‐making. Indeed, patients might show resistance regarding sustainable choices that could directly affect their treatment and would compromise clinical effectiveness, which was also highlighted in the Dutch national survey and a 2024 survey study among gynaecological patients of two outpatient clinics in the Netherlands [11, 16]. However, the ethical obligation to reduce greenhouse gas emissions suggests that all social sectors – including healthcare – ought to be more sustainable. This prompts an exploration of if and when environmental concerns should be integrated into clinical decision‐making and whether patients believe that individual needs should take precedence over environmental considerations or if there are circumstances in which trade‐offs may be ethical and clinically acceptable.

While economic considerations, such as healthcare costs, are increasingly studied in regard to shared decision‐making processes, research on patient attitudes towards integrating environmental outcomes into healthcare decision‐making remains scarce [13, 17, 18, 19, 20]. Qualitative studies are needed to gain a more profound understanding of patient values, preferences and the acceptability of discussing environmental outcomes of healthcare practices within patient–provider consultations. Gaining insight into patient attitudes can shape how environmental information is incorporated into consultations, enabling meaningful patient involvement. This approach would not only make clinical practice more sustainable but also safeguard patient wellbeing amidst the ongoing ecological crisis, which increasingly impacts both health and access to care. This interview study serves as an in‐depth follow‐up of a survey study on patients' perspectives on environmental sustainability within healthcare settings [16]. Specifically, this study aims to elucidate patients' attitudes regarding the provision of environmental information in healthcare decision‐making and to identify preferred approaches for integrating these considerations into patient–provider consultations. This focus on communication strategies within consultations ensures that patient perspectives directly inform the evolving approach to sustainability in healthcare decision‐making while upholding principles of clinical ethics.

## 2 Methods

We performed qualitative research conducting semistructured interviews with patients of two outpatient clinics in the Netherlands. To elicit in‐depth responses and the nuances of patients' experiences regarding environmental sustainability in healthcare settings, we decided to organize one‐on‐one interviews. The Ethics Review Board of Amsterdam University Medical Centre (Amsterdam UMC) concluded that the research did not fall within the scope of the Medical Research Act with People (METC application number 2024.0027, date of approval 31 January 2024). We adhered to the Standards for Reporting Qualitative Research (Table S1) [21].

### 2.1 Study Design and Setting

The present interview study served as an in‐depth follow‐up to a prior survey study in obstetrics and gynaecology, concerning patients' levels of climate‐related worry, knowledge and preferences regarding the incorporation of environmental sustainability into healthcare practices. Details on this previous study are published elsewhere [16]. The follow‐up interview study was conducted between February and May 2024 at two outpatient clinics of Amsterdam UMC, a tertiary hospital in the Netherlands.

### 2.2 Participants and Recruitment

Participants were patients aged 18 years or older who had visited at least one of the following outpatient departments at Amsterdam UMC in the past 2 years (2023 or 2024): fertility, obstetrics, benign gynaecology, gynaecological oncology and prenatal diagnostics. We recruited patients through purposive sampling via email based on their responses to the previously published questionnaire, which included their written informed consent to be contacted for potential participation in a follow‐up study. To ensure a wide range of perspectives, we first invited a total of 35 patients from the highest, middle and lowest climate‐friendly healthcare perspective score groups based on the previous questionnaire study. These scores were based on six questions about individual and healthcare system contributions to climate change, as well as openness to potential climate change mitigation strategies, such as online consultations and paying extra for climate‐friendly health insurance. In case of non‐responses, we emailed the remaining eligible participants on the list. In total, we emailed 65 patients, of whom 9 agreed to participate. After the first round of interviews, we concluded that at least three additional interviews were needed to capture perspectives from patients less concerned with environmental sustainability [22]. Therefore, two physicians of our research team purposefully invited nine more patients, stratified by subdepartment, and indicated a lower interest in environmental issues, five of whom agreed to participate. All patients provided written informed consent and were asked to complete the previously published questionnaire before the interview to assess their climate‐friendly healthcare perspective scores. We excluded patients who did not speak English or Dutch. Participants received a 25 Euros voucher for their participation. The study team debriefed and analysed transcripts after the final interviews and continued data collection until no new substantial insights and patterns of shared meaning emerged from the data [23].

### 2.3 Data Generation

The research team included several experienced qualitative researchers (JWMA, DSK) who supervised and mentored junior researchers (ESC and FG) during the data generation and analysis process. All interviews were conducted by a junior researcher (ESC) who was trained in conducting qualitative research. During this training, researchers reflected on their own preconceived ideas and biases regarding the study topic and received guidance on how to manage these biases consciously during data generation and analysis (for a comprehensive reflexivity statement, please refer to Supplementary Information). The interviewer (ESC) had no prior or prospective (therapeutic) relationship with any of the patients. The research team developed an interview guide to facilitate the interviews based on literature and results of the previously published questionnaire study (Supporting Information ‘Interview guide’). The guide began with a reflection on the questionnaire responses and deliberately excluded background information on environmental sustainability to obtain a representative insight into patients' knowledge and experiences on the subject. Throughout the interview, patients were invited to reflect on specific examples, including their (environmental) considerations regarding the use of reusable and disposable specula, as well as treatment options for heavy menstrual bleeding, cancer or contraception. The interview guide was tested within the research team before the interviews took place and was iteratively refined after the first three interviews. Participants were interviewed either online or at a location of their choice. Interviews were recorded using a voice recorder or via Microsoft Teams, and the recordings were transcribed verbatim using Microsoft Teams' transcription function. Audio recordings were deleted after transcription, and all identifiers were removed from the transcript. The transcript was shared with the participants following each interview upon request, but no comments or corrections were received.

### 2.4 Qualitative Analysis

Qualitative coding and analysis were conducted using MAXQDA 2022 (VERBI Software, 2021) [24]. The analysis team consisted of two qualitative research experts (JWMA and DSK) and two junior researchers (ESC and FG). Our methodological approach followed reflexive thematic analysis (TA) as described by Braun and Clarke. We adopted a constructionist epistemology, focusing on the interpretative nature of complex qualitative data to identify key patterns of shared meaning in integrating environmental sustainability into healthcare decision‐making and patient–provider consultations [23, 25, 26]. Therefore, we employed a recursive reflexive TA process guided by six phases [26]. First, we familiarized ourselves with the data by listening to the audio‐recordings, reading the transcripts and reviewing the analytical field notes written by the interviewer following each interview. Second, to ensure multiple and rich interpretations, two researchers (ESC and FG) independently coded the transcripts line‐by‐line in two rounds of initial coding, both at a semantic (explicit) and latent (conceptual) level. Following this, two researchers (ESC and FG) compared and discussed their codes with two experienced researchers (JWMA and DSK). We employed a predominantly inductive approach, meaning data was open‐coded to allow for a data‐driven analysis that reflected the meaning conveyed by the participants [27]. However, we also applied some degree of deductive analysis based on the interview guide, ensuring that the open‐coding contributed to themes that aligned with the research questions. Ultimately, we collated all codes and developed a shared codebook after reaching a consensus within the research team. Third, we generated initial themes and subthemes from clusters of codes and reviewed the viability of each potential theme with the research team. Fourth, we refined the themes as patterns of shared meaning and compared them with data extracts. During phase five, we further refined and named the final themes. In the sixth and final phase, we wrote up the report and contextualized compelling data extracts. Notably, we moved back and forth between these phases throughout the analysis process.

## 3 Results

A total of 14 patients from 5 gynaecological subdepartments were interviewed. All participants completed the previously published questionnaire, which included questions on demographics and climate‐friendly healthcare perspectives (Table 1) [16]. The median age was 41 years (range 31–68 years), and all participants identified as women. Most participants worried about the consequences of climate change and agreed that healthcare contributes to CO_2_‐emissions and thus to climate change.

**Table 1:** Patient characteristics (*n* = 14).

Participant number	Department	Age	Educational background	Ethnicity*	I am worried about the consequences of climate change	Healthcare contributes to CO_2_‐emissions and thus to climate change	Climate friendly healthcare perspectives score**
1	Benign gynaecology	61	University of applied sciences	None provided	Totally agree	Totally agree	25
2	Obstetrics	39	University	Dutch	Totally agree	Totally agree	24
3	Reproductive medicine	32	University	Dutch, French	Totally agree	Agree	24
4	Oncology	66	University of applied sciences	Dutch, European	Totally agree	Agree	20
5	Benign gynaecology	40	Secondary school	None provided	Totally disagree	Disagree	11
6	Prenatal diagnostics	33	University	Dutch	Agree	Agree	21
7	Reproductive medicine	34	University of applied sciences	Dutch	Neutral	Neutral	18
8	Benign gynaecology	34	University	Rwandan, African	Neutral	Neutral	17
9	Reproductive medicine	41	University	Dutch	Totally agree	Agree	22
10	Benign gynaecology	43	University of applied sciences	Surinamese, Dutch	Agree	Agree	19
11	Benign gynaecology	51	University of applied sciences	Dutch	Agree	Agree	20
12	Oncology	68	University of applied sciences	Dutch	Agree	Agree	17
13	Benign gynaecology	50	University of applied sciences	Surinamese, Dutch	Agree	Neutral	15
14	Obstetrics	39	University	Russian, Dutch	Agree	Neutral	13

* Self‐identified ethnicity, multiple answers possible.

** Questionnaire score. Higher score indicating more climate‐friendly perspectives (minimal score of 6, maximal score of 30).

**Figure 1:**
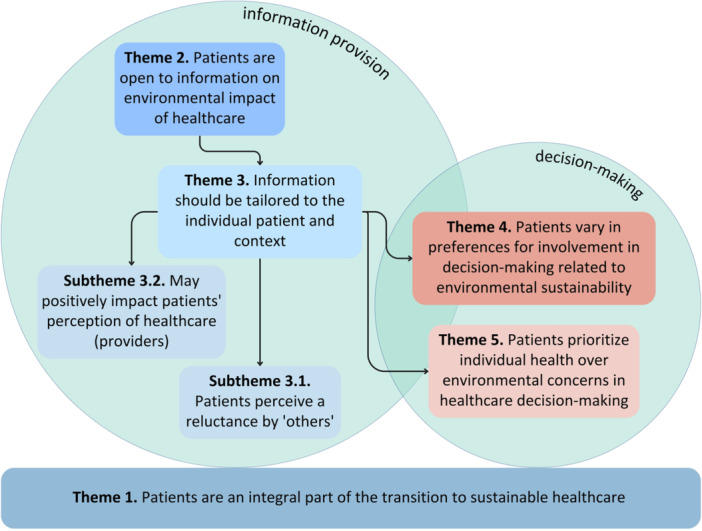
Visualisation of themes and subthemes.

The interviews averaged 49 min in duration (range 40–83 min) and were conducted between February and May 2024. None of the participants had previously received information on the environmental impact of healthcare within patient–provider interactions. Based on their own experiences as patients, most participants highlighted the substantial waste produced by the healthcare sector and the waste associated with leftover medication. Five main themes and two subthemes were identified as visualised in Figure 1. The first theme provided the foundation for the other themes. Themes two and three were associated with information provision, whereas themes four and five addressed decision‐making within patient–provider interactions.

### 3.1 Patients Are an Integral Part of the Transition to Sustainable Healthcare

Participants believed that sustainable healthcare aligns with the role and responsibility of the healthcare sector and referred to patients as an integral part of it. Participants recognized their individual role as citizens in society and believed that everyone should contribute to sustainability where possible, with no exception for healthcare settings. However, participants believed that individual patients play a smaller role compared to other stakeholders and acknowledged the significant responsibility of the government, industries, hospitals and the healthcare sector as a whole to achieve systemic change.

The hospital management … or rather, the leadership of the hospital is very responsible for how they communicate this to their staff. And only after that does the client come in. Because, yes, it may sound blunt, but a hospital is a business, and if you can hold Shell or another company accountable, why can't a hospital be held accountable just because it deals with human life and well‐being? Yes, precisely because you are dealing with life and the well‐being of people, shouldn't you set a good example?

– Participant 1

Every individual has a responsibility in healthcare, for everything around you. And that also includes the care you will receive. The use of healthcare, I simply see healthcare as a product. […] Yes, every individual is responsible for that.

– Participant 4

How wonderful would it be to connect care, so to speak, care for yourself with care for the world. […] Healthcare might not be the first industry people look at to reduce emissions. If healthcare does manage to do that, it almost has a heroic quality. I find that very admirable. Also because it is, in fact, completely in line with the nature of the business; it is fully aligned with it.

– Participant 11

### 3.2 Patients Are Open to Information on Environmental Impact of Healthcare

Participants expressed to find it important to be well‐informed on the environmental impact of healthcare practices. A key point made by participants was that information dissemination on environmental impact would raise awareness among patients, which they felt may encourage environmental actions beyond the healthcare setting. Normalizing discussions about environmental impact within healthcare settings was recognized as a gradual and necessary process. However, participants noted that these conversations are not yet widely accepted or common, and that the society as a whole is still in the early stages of transitioning towards sustainable practices. This was illustrated by the fact that none of the participants had prior experience in receiving information on the environmental impact of healthcare practices within healthcare settings. A lack of knowledge and awareness among patients regarding the environmental impact of healthcare was seen as a barrier to starting conversations about sustainability. However, it was also presented as an opportunity for change, provided that efforts are made to improve patients' understanding through education, such as hospital campaigns.

I would like it to get a bit of information, so that, yes, it can also help me to switch my perspective. Maybe I should also change my life a bit, my way of living, to participate in the climate and to think about it, so I think that's good. Knowing the information. Knowing information is always good.

– Participant 5

Well, I think you can involve them in that, indeed giving them a choice and perhaps also creating awareness among people. Because, now that you mention it, I realize that I know they [vaginal specula] are made of plastic, but I hadn't thought at all about the fact that they are being thrown away. So, yes, I do find that an issue.

– Participant 7

Yes, I think it's quite important. But of course, the most important thing is providing good care. However, since we all need to do our part, it's not a bad idea if the client or patient is aware of it. Although it's a difficult topic, because it's not the primary goal, but the patient still needs to be well‐informed, so why not?

– Participant 6

### 3.3 Information on Environmental Impact Should Be Tailored to the Individual Patient and Context

Participants expressed diverse preferences regarding the context of information provision on environmental outcomes within healthcare settings. Some participants believed environmental topics should always be mentioned, allowing the patient to choose whether to engage with the information. In contrast, some believed it was essential to first assess a patient's interest in the environmental impact of healthcare decisions. All mentioned facilitators and barriers for integrating environmental outcomes in healthcare settings. For instance, participants noted that emergency settings are not conducive to discussions on the environmental sustainability of treatments, whereas chronic disease management with continuity of care provides a more suitable context for such conversations. Other identified facilitators and barriers included patient's health literacy, time constraints, severity of disease and family involvement (involvement of significant others). Patients varied in preferences regarding strategies to communicate environmental information, which included but were not limited to conversations in the consultation room, via websites, package inserts, patient information sheets or patient campaigns.

Often, patients receive a lot of information, and some people can process it well and may want even more control, while others would prefer that decisions are made for them. So, if that information is available for those who find it useful and important, I think that's great and beneficial, and the doctor can discuss it with them. But I also think there is a large group of people for whom considering this information is simply not feasible.

– Participant 2

I think as long as it's not confrontational conversations, for example. If you have a calm discussion and, you know, if you're having a major conversation about a miscarriage or something like that, then I wouldn't do it as a doctor. But if, for example, I've been for a check‐up and everything is just fine and you haven't had that conversation yet, then it could be a good moment for the doctor to say, well, maybe this is a good time. I think a doctor can also assess well in their profession what is a good time to discuss that.

– Participant 7

It depends on the patient condition. When someone is an emergency condition, I don't personally want a doctor to come and start talking about climate change and environmental impact. Except if it's something that is going to affect me in that emergency situation. But maybe after some time, or also if it's part of the treatment that is going to be used, maybe it can be emphasized on, but in many cases, let's say mostly in emergent situations, personally I don't find it really practical, yeah.

– Participant 8

#### 3.3.1 Informing Patients on Environmental Impact May Positively Impact Their Perception of Healthcare (Providers)

Consistent with the finding that participants considered the need to transition to sustainable healthcare, participants expressed support for promoting information provision on environmental sustainability by healthcare professionals. Participants stated that information about environmental impact would enhance positive perceptions when provided under appropriate conditions (as described by theme 3). Indeed, inappropriate timing or delivery of information were thought to possibly evoke negative feelings or feelings of mistrust; healthcare professionals' communication skills were deemed to be crucial.

Yes, I think it is important that he [the healthcare professional] does provide the information, because that shows me that he thinks about it, he also wants to help me think about it and make the decision about the choice I am going to make. Whether it affects the climate or not and my health, so I think it is good, and he also shows me that he has knowledge about what all of that means, climate change, together with care, yes.

– Participant 5

It shows commitment. And thus, probably also commitment towards me. I think it is important to see that a doctor is involved with the patients, and not only with the patients, but also with potential patients. I think that if, for instance, a treatment might be detrimental to the environment like we talked about before, that also means there could be patients affected by it. So yes, it is definitely good that they are addressing this.

– Participant 13

#### 3.3.2 Patients Perceive a Reluctance by ‘Others’

While participants in this study were open to information on environmental sustainability, most noted that ‘other’ patients may be reluctant to engage with environmental information, who may find these conversations confrontational. This perception was primarily shaped by general experiences, such as the reactions of family, friends or politicians to environmental and climate issues, rather than by direct experiences within healthcare settings.

Yeah, I would say it's good to know [the environmental impact of care] for me. But I also can imagine that some people would be strongly against it, because they don't want to feel kind of guilty for their choice.

– Participant 14

I find that [the information on environmental impact] important, but at the same time, I know that there are many people who find it less important or not important at all. However, I would find that information very interesting and might eventually include it in my overall consideration. So that it is actually additional to the elements you currently consider when deciding whether or not to undergo a particular treatment.

– Participant 10

### 3.4 Patients Vary in Preferences for Involvement in Decision‐Making Related to Environmental Sustainability

Some participants preferred to be involved in decision‐making related to environmental sustainability and to have the opportunity to consider information on environmental outcomes when making treatment decisions. Others preferred decision‐making at higher levels in the healthcare system, for example, by healthcare professionals or at an institutional level. Participants recognized that certain decisions, such as those related to disposable or reusable speculum use, should be made by experts in the field or guided by national healthcare guidelines. This was supported by participants who expected to receive effective care with minimal environmental impact without being overly burdened by decisions that could be made at higher organizational levels. At the same time, participants expressed a desire for increased patient involvement when the personal impact of a procedure or treatment was deemed significant and multiple characteristics of care could be considered, such as extended medication regimens or contraceptive options. In case of treatments with similar effectiveness across options, participants' preferences varied. Some viewed this as an ideal opportunity for patient involvement in environmentally sustainable decisions to create awareness and to be well‐informed of all options, while others questioned why healthcare professionals would not just provide the option with the lowest environmental impact without burdening the patient with information about alternatives. Few participants also recognized the importance of discussing the environmental impact of healthcare when no alternative medical treatment is available to raise the patient's awareness of the environmental impact of healthcare. Others expanded on this by noting that alternatives always exist, such as opting for no treatment, clinical surveillance or palliative care.

When someone enters a hospital, they already have a lot on their mind, and I think that the part where the hospital can relieve them should be tackled very thoroughly by the medical staff, the hospital organization, and the management department. What remains can still be communicated to the client and provided with information.

– Participant 1

I would like to be able to choose, like knowing exactly what are the consequences for environment, but also if it's a treatment, like the effectiveness, like to compare. So really to have all the information to make my choice about what are the side effects or how effective it will be, but also how this one will be more eco‐friendly than this one. And really, to make up my mind by myself with medical advice. Yeah, I would appreciate that.

– Participant 3

I find the whole culture of consultations in healthcare quite annoying. I go to the healthcare professional, so I expect them to have done the research and present me with the best option. So, if there is an option that is just as efficient and works just as well, but is better for the environment, I expect my healthcare provider to make that choice.

– Participant 9

### 3.5 Patients Prioritize Individual Health Over Environmental Concerns in Healthcare Decision‐Making

Many participants were more concerned with their individual health than with the environment within healthcare settings. They considered ‘getting better’ to be the primary goal of seeking healthcare and, therefore, to be more important than environmental concerns. Some participants expressed feelings of ‘ego‐centrism’ and ‘selfishness’ regarding this prioritisation. Although only one participant spoke of potential feelings of guilt, there appeared to be some degree of moral friction because multiple participants did offer their apologies for superseding their individual needs over their environmental worries. Even though the effectiveness of medicines and surgeries to treat diseases were highly valued, participants reported that decision‐making regarding sustainable healthcare is highly personal and context‐specific, with possible trade‐offs involving effectiveness, comfort, side‐effects and environmental impact. For example, some participants were willing to accept less comfort or more side‐effects to receive the most sustainable care, for example, by choosing a different form of treatment administration or by opting for a reusable speculum.

I think that, initially, patients just want good care and to get better. How that happens, I don't think they are really focused on that at first. […] I think I would actually be more concerned about what they are going to do, and not so much about what that means for the climate. Maybe that's selfish.

– Participant 13

Maybe it's a bit selfish as well, because I know like sustainability is for everybody, but I feel if you have cancer or if you have like a very severe disease, maybe your first, uh, care is like how much it will help me with as less side effects as possible. That's how I think I would react.

– Participant 3

If they say that this one is better for the environment, but the other one works better, then of course, if I have cancer, I would choose the option that helps me the most. Honestly, at that moment, I don't think it matters if it's worse for the environment. I might still want information about it, and it could also make a difference whether I'm 86 or 42.

– Participant 9

## 4 Discussion

Our study revealed that patients are open to receiving environmental information in healthcare settings, recognizing that both the healthcare sector and individuals have a shared responsibility in adopting sustainable practices. While patients acknowledged the need for environmental awareness, they expressed concerns about potential confrontations with others who might not be as receptive. Despite this, they emphasized the importance of personalized care and context‐sensitive conversations on environmental impact. Patients prioritized individual health over environmental considerations, but some would accept environmentally friendly healthcare practices that involve reduced comfort or increased side effects, depending on the context.

These findings align with other studies that show patients' increasing interest in environmentally sustainable healthcare [11, 13, 16, 28, 29]. While concerns have been raised in the literature about the risk of potentially compromising the physician–patient relationship when mentioning environmental topics, our study highlights the potential benefits and positive impact these conversations could have when initiated under adequate conditions [13, 30]. In fact, one patient even perceived environmental conversations within the healthcare context as ‘heroic’, and another found it compelling if healthcare professionals would consider not only the patient in front of them, but also future generations and broader societal implications. These findings are consistent with a larger focus group study in the United States, that showed that both patients and physicians were interested in participating in climate and health conversations, and agreed that climate discussions in healthcare settings could increase awareness [12]. The latter study also found that physicians underestimated the interest and willingness of patients to engage in environmental conversations – a perception that aligns with our findings, showing that patients underestimated the interest of other patients. These studies add an important normative dimension to sustainable healthcare, defeating arguments that resource conservation is unimportant or irrelevant in clinical care.

A large study published in 2024 investigated this phenomenon known as the ‘perception gap’ between actual and perceived support for climate action among 130,000 people across 125 countries [31]. People significantly underestimated the willingness of their fellow citizens to contribute to climate action. These systematic gaps between perception and reality can form an obstacle to engage in climate action, thereby confirming the negative beliefs held by others. Our study showed a positive perception of patients with regard to receptiveness to environmental information. Therefore, the study should be considered an effort to correct any misperceptions by healthcare professionals and patients themselves regarding patients' positive attitudes towards transitioning to environmentally sustainable healthcare. Instead of echoing the concerns of a minority that opposes any form of environmental conversations, our study helps communicate that patients are worried about the consequences of climate change and would find it both acceptable and desirable to incorporate environmental considerations into healthcare decision‐making in a patient‐centred context. As such, clinical ethics would accept discussions on climate change and the environmental impact of care under the umbrella of informed consent, patient autonomy and patient preferences [32].

This support for sustainability considerations within healthcare does not come without caveats: patients outline clear conditions under which these types of conversations are appropriate and when these are not. For example, patients highlighted that discussing the environmental impact of healthcare does not have a place in emergency settings that require immediate clinical action, when there are time constraints, and when there is lowered patient decisional capacity in case of low health literacy or experienced anxiety, stress or pain. This is consistent with widely held views of information provision in patient–provider encounters, which truncate the quantity and kind information given [33]. On the other hand, patients reported that the healthcare sector should strive to implement sustainable practices whenever feasible (e.g., purchasing green energy and using as few disposables as possible) and present options to patients in situations of clinical uncertainty or preference‐sensitive equipoise (e.g., when deciding which contraceptive to use). Patients highlighted that elective treatments for long‐term non‐life‐threatening conditions are particularly suitable for discussing environmental impact, such as in the case of (surgical) treatment for heavy menstrual bleeding. Interestingly, these facilitators and barriers resemble those reported in the context of shared decision‐making [34, 35, 36]. Some of the principles of shared decision‐making could therefore usefully guide environmental communication within healthcare settings. The core of shared decision‐making lies in the view that individuals should be aware of all relevant options and their characteristics, and that their personal perspectives and preferences are taken into consideration when determining choices [37]. Similarly, as demonstrated in our study on discussing environmental outcomes, the degree to which patients wish to be involved in decision‐making, as well as their desire to become informed about relevant information, will vary. Our findings suggest that environmental sustainability should be normalized among other forms of health information provision in non‐emergency settings. The sharing of this information, as well as involving patients in related decision‐making, should always be tailored to the individual patient and the specific context. In the end, this will enable patients to consider how environmental impact, along with other factors, affects their treatment choices and will foster awareness beyond the consultation room. Moreover, patients reported that receiving information on environmental impact in an adequate manner could positively influence their perceptions of the care they receive, as it demonstrates healthcare's commitment to and concern for public health.

There are some important considerations regarding the inclusion of environmental impact within shared decision‐making frameworks. First, patients generally reported that the impact of environmental outcomes on decision‐making is expected to be minimal because individual health is prioritised over sustainability. Rather than emphasizing the need of healthcare decisions alone, the focus could be redirected toward facilitating patient discussions about environmental impact (e.g., using patient conversation aids instead of patient decision aids), which patients in our study identified as particularly valuable. Second, ethical challenges related to environmental conversations in healthcare settings should also be taken into account, a topic we have explored elsewhere [38]. In short, these discussions address balancing the inclusion of environmental impact information with the need to avoid moral coercion and to preserve patient autonomy [30, 38]. The findings of our current study, however, suggest that we should not assume these topics have no place in patient consultations. Our study underscores the importance of integrating sustainability into clinical practice, aligning with bioethical principles and the expectations and goals of patients. Similarly, increased ethical concerns about financial burden (so‐called ‘financial toxicity’) within healthcare have translated into recommendations to include information about pricing and costs of healthcare in shared decision‐making, increasing the frequency of cost conversations about treatment options [39, 40, 41]. While notable differences exist between personal healthcare expenses and environmental ‘costs’ such as negative public health effects, certain parallels are evident in the literature: studies have found that some patients may feel more comfortable than healthcare professionals when discussing these environmental and economic costs and may support the inclusion of such considerations, whether addressed by physicians or as part of their own decision‐making responsibilities [12, 42]. Third, questions arise regarding whether we should consider environmental information within the same context as shared decision‐making, given that it is a public health issue where wider societal interests may supersede individual preferences, similar to the case with vaccinations [35]. As we confront the environmental crisis, there is a need to establish new standards for quality of care. This necessitates centring medical expertise while also incorporating patient perspectives on a fundamental level through proactive co‐design of services. For example, we could take patients' environmental considerations into account when designing medical products like specula rather than soliciting their opinions after implementation in clinical practice, which may leave patients feeling abandoned when making complex decisions [43]. Future research should examine the role of environmental sustainability within the context of shared decision‐making, preferably in real‐life situations that are applicable to the target population. Lastly, the provision of information on environmental impact should not exacerbate health inequities or evoke feelings of guilt. Clinicians should be aware that feelings of guilt might be higher in marginalized populations, particularly women, who are socialized to be self‐sacrificial [44].

### 4.1 Strengths and Limitations

Through this in‐depth interview study, we elicited patients' attitudes towards incorporating the environmental impact into healthcare decision‐making. While qualitative research does not aim for generalizable results, our focus was to address specific research questions through rich, meaningful and complex narratives, as suggested by Braun and Clarke [22]. Therefore, we captured diverse patient experiences, encompassing patients undergoing pregnancy check‐ups, as well as fertility, benign gynaecological and oncology treatments.

This study presents several limitations. First, none of the patients had prior experience with receiving environmental information in a healthcare setting. As a result, while these patients all reflected on their values regarding sustainability, their responses may not fully represent reactions grounded in real‐life scenarios. To test some of the hypotheses formulated by the patients, future research should be conducted in an empirical setting. This would involve providing some patients with information about environmental impacts while withholding it from others, followed by interviews to assess their experiences and perceptions regarding the intervention. Second, we selected only a subset of patients, specifically women visiting two gynaecological outpatient clinics in the Netherlands. Patients identifying as women might be more actively engaged in issues of sustainability; research indicates that women are more likely to exhibit pro‐environmental attitudes compared to men [45, 46]. Further research is needed to determine whether the shared patterns of meaning identified in our study are equally applicable to men. However, similar narratives were also identified in an American context involving men and women, and the responses to and median scores of the pre‐interview questionnaire of this study were consistent with the data from our larger survey study among 274 patients (both men and women), suggesting some potential for transferability to broader patient groups and international contexts. Nonetheless, to provide an even wider range of patient perceptions, this interview study might be repeated in other, less educated and non‐gynaecological patient groups. Finally, we included both patients who were actively receiving treatment at the hospital and those who had completed their treatment within the past 2 years. It could be hypothesized that some patients who were no longer undergoing treatment may have assigned a lower value to treatment options and a higher value to environmental considerations, and vice versa.

### 4.2 Implications and Future Research

Patients express a need for information on the environmental impact of healthcare and highlight the responsibility of healthcare institutions and providers to offer the most sustainable care whenever feasible. To achieve this, the environmental impact of care must first be quantified, compared and improved wherever possible. Our results emphasize the need to conduct environmental impact assessments, with patients indicating particular interest in non‐acute, elective settings involving non‐life‐threatening conditions, where several treatment options are available that are relatively comparable in clinical outcomes. Therefore, these care pathways should be prioritized when quantifying environmental impact to provide patients with relevant information. Additionally, to prevent patients from feeling abandoned while burdening them with complex environmental trade‐offs, policymakers and insurers should come up with plans to prioritize sustainable, clinically effective healthcare, incorporating environmental impact into healthcare technology assessments as an integral part of the quality of care. Individual patient–provider decisions should only be additive in the context of this system‐level change.

In both healthcare and everyday life, people make decisions by balancing values. Without eliciting patient values, healthcare professionals may overlook or disregard a patient's environmental concerns, potentially leading to an inaccurate perception of disinterest and limiting patient autonomy. By providing environmental information within the appropriate context and in the appropriate tone – with the help of tools such as patient conversation aids or information leaflets – patients can be empowered to consider environmental values in their decision‐making. Naturally, if someone is not concerned about the environment at that moment, the conversation may shift to focus on other values that are more important to them. To enable these ethically appropriate conversations in clinical settings, healthcare professionals must also be engaged and feel comfortable addressing these topics. Future research should investigate healthcare professionals' attitudes regarding the integration of environmental impact into patient–provider encounters.

## 5 Conclusion

This study demonstrates that patients are open to receiving environmental information as part of patient‐centred care, specifically in non‐acute elective settings. Our findings suggest that with the right approach, healthcare professionals can help bridge the gap between patient attitudes and perceived support for sustainability initiatives. By integrating patient preferences into care and ensuring that environmental considerations are introduced in a personalized and context‐appropriate manner, healthcare can foster greater support for sustainable practices, accelerating systemic changes in the sector.

## Author Contributions

**Eva S. Cohen:** conceptualization, investigation, funding acquisition, writing–original draft, methodology, writing–review and editing, formal analysis, data curation, visualization, project administration, validation. **Dionne S. Kringos:** conceptualization, investigation, funding acquisition, writing–review and editing, methodology, data curation, supervision, validation, resources. **Fleur Grandiek:** investigation, writing–review and editing, data curation. **Lisanne H. J. A. Kouwenberg:** visualization, data curation, conceptualization, methodology, writing–review and editing. **Nicolaas H. Sperna Weiland:** conceptualization, funding acquisition, writing–review and editing, methodology. **Cristina Richie:** conceptualization, writing–review and editing. **Wouter. J. K. Hehenkamp:** supervision, conceptualization, investigation, funding acquisition, writing–review and editing, data curation, methodology, validation, resources. **Johanna W. M. Aarts:** conceptualization, funding acquisition, investigation, writing–review and editing, supervision, methodology, data curation, validation, resources.

## Ethics Statement

The Ethics Review Board of Amsterdam University Medical Centre (Amsterdam UMC) concluded that the research did not fall within the scope of the Medical Research Act with People (METC application number 2024.0027, date of approval 31 January 2024).

## Conflicts of Interest

The authors declare no conflicts of interest.

## Supporting information

Supporting information.

## Data Availability

The data that support the findings of this study are available from the corresponding author upon reasonable request.
