# Supplementary material for: Patients' Attitudes Towards Integrating Environmental Sustainability Into Healthcare Decision‐Making: An Interview Study
Source: Health Expect. 2025 Jan 19;28(1):e70155. doi: 10.1111/hex.70155 (PMC11743189; doi:10.1111/hex.70155)
Supplement: Supplementary file 1 — Supporting information. [file HEX-28-e70155-s001.docx]

**Patients’ attitudes towards integrating environmental sustainability into healthcare decision-making: an interview study**

**Supplementary Information**

**Table of contents**

1. Interview guide

2. Researcher reflexivity statement

3. Supplementary Table 1: Standards for Reporting Qualitative Research Checklist

## Interview guide

Part 1. General information about the interview

- Personal introduction
- Brief explanation of the research and clarification of terminology used
- Purpose of the interview
- Expected duration of the interview
- Consent to audio recording of the interview
- Information on anonymizing interview data and the possibility of receiving the transcript

Part 2. Reflection on answers from previously completed questionnaire

- Experience with healthcare: brief explanation of past or current treatment trajectory
- Concerns about climate change
- Knowledge about climate change, discussing concept environmental sustainability
- Personal engagement with the topic of environmental sustainability
- Relationship between sustainability and healthcare, self-reported examples of the environmental impact of healthcare and possible mitigation measures
- Integrating environmental impact in shared decision-making
- Identifying examples of sustainable choices in gynaecological care: specula (disposable versus reusable), contraception, and treatment of heavy menstrual bleeding

Part 3. Information about the environmental impact of healthcare

- How important do you consider it that healthcare professionals provide you with information about the environmental impact of healthcare?
  - Does this differ from other outcomes you are informed about in healthcare, and why?
  - Have you ever received information from a healthcare provider about the environmental impact of healthcare or a treatment?
- How important do you consider it to have a choice as a patient regarding the environmental impact of your healthcare?
  - About which aspects of a treatment would you like to have a choice regarding the environmental impact of your healthcare? For example: the use of sustainable materials or the use of sustainable medication?
  - When should the healthcare sector or the hospital decide to offer only the most environmentally sustainable option, and when should the patient be involved in healthcare decision-making related to environmental sustainability?

Part 4. Role of the patient in the environmentally sustainable transition of healthcare

- What role do you see yourself playing as an individual patient in addressing societal issues related to healthcare, such as the ecological crisis?
  - No role at all? A significant role?
  - How does this relate to the role of the healthcare provider, the hospital, health insurers, pharmaceutical companies, etc.?
- Who should make choices in the healthcare sector to make healthcare more environmentally sustainable?
  - For example, doctors, individual patients, patient associations, health insurers, hospital management, etc.
  - Why those group(s)?

Part 5. Provision of information and considerations of the environmental impact of treatments

- For what kind of treatments would you prefer to be informed and for what kind of treatments would you not prefer to be informed about the environmental impact of treatment alternatives?
  - What characteristics make a treatment or care pathway suitable or unsuitable for discussing environmental sustainability with patients? Examples: treatment of heavy menstrual bleeding, cancer treatment, choice of contraception, fertility treatment
  - Is it also desirable to receive information about environmental impact when there is no alternative treatment available?
- In what way do you think information about the environmental impact of a treatment may affect your choice of treatment?
  - What would be helpful to make an environmentally sustainable choice?
  - What trade-offs are involved, and how do you weigh environmental sustainability against other characteristics of a treatment (for example: effectiveness, safety, comfort)?

Part 6. Communication and patient-provider relationship

- In what way would you prefer to be provided with information about the environmental impact of healthcare decisions?
  - For example: through a leaflet, in a conversation with a healthcare professional, in advance, in a follow-up appointment, through a website, etc.
- Imagine that your healthcare provider discusses the following characteristics of two treatments (medicine A and B): 1) side effects; 2) how well the medicines work (effectiveness); 3) the waste and CO_2_-emissions produced during their production and use. How would this influence your feelings toward the healthcare provider and the care you receive?
- How might discussions about environmental sustainability by healthcare providers affect your trust in healthcare and in healthcare providers?

**Researcher reflexivity statement (first author)**

As a junior researcher, I (ESC) was trained in qualitative research and conducted all interviews with the participants. I recognize data generation and analyzing as an inherently subjective process, prompting this reflexivity paragraph to elucidate subjectivity.

As a cis- and white woman raised in a left-wing urban family, I am actively engaged in promoting environmental initiatives and in advocating for equitable climate policies in and beyond healthcare. My research project focuses on greening healthcare practices, with an emphasis on prioritizing the patient perspective. In pursuit of patient empowerment, it was important to remain receptive to diverse patient perspectives during the interview conduction, even if these diverged from my own views on the sustainable transition in healthcare systems. This awareness was essential to foster an environment during interviews where opposing viewpoints could be expressed, allowing for further exploration of patients’ attitudes. Moreover, I had no history or prospective (therapeutic) relation with any of the participants as a medical doctor, something I made explicit at the start of each interview.

As a researcher I was also trained at the Centre for Sustainable Healthcare of the Amsterdam UMC, which included a course on conducting "climate conversations," emphasizing the importance of listening to individuals with differing opinions and accepting ambivalence. Furthermore, recognizing that diverse perspectives are crucial for accurately addressing the research question, we as a research team actively sought and incorporated such perspectives in our purposive sampling approach.

|  | **Supplementary Table 1: Standards for Reporting Qualitative Research (SRQR) Checklist** | | |
| --- | --- | --- | --- |
|  | |  |  |
|  |  | | **Page no(s).** |
| **Title and abstract** | | |  |
|  | **Title** - Concise description of the nature and topic of the study Identifying the study as qualitative or indicating the approach (e.g., ethnography, grounded theory) or data collection methods (e.g., interview, focus group) is recommended | | Title page |
|  | **Abstract** - Summary of key elements of the study using the abstract format of the intended publication; typically includes background, purpose, methods, results, and conclusions | | 1 |
|  |  | |  |
| **Introduction** | | |  |
|  | **Problem formulation** - Description and significance of the problem/phenomenon studied; review of relevant theory and empirical work; problem statement | | 3-4 |
|  | **Purpose or research questio**n - Purpose of the study and specific objectives or questions | | 4 |
|  |  | |  |
| **Methods** | | |  |
|  | **Qualitative approach and research paradigm** - Qualitative approach (e.g., ethnography, grounded theory, case study, phenomenology, narrative research) and guiding theory if appropriate; identifying the research paradigm (e.g., postpositivist, constructivist/ interpretivist) is also recommended; rationale | | 6-7 |
|  | **Researcher characteristics and reflexivity** - Researchers’ characteristics that may influence the research, including personal attributes, qualifications/experience, relationship with participants, assumptions, and/or presuppositions; potential or actual interaction between researchers’ characteristics and the research questions, approach, methods, results, and/or transferability | | Supplement:  Researcher reflexivity statement |
|  | **Context** - Setting/site and salient contextual factors; rationale | | 5-6 |
|  | **Sampling strategy** - How and why research participants, documents, or events were selected; criteria for deciding when no further sampling was necessary (e.g., sampling saturation); rationale | | 5 |
|  | **Ethical issues pertaining to human subjects** - Documentation of approval by an appropriate ethics review board and participant consent, or explanation for lack thereof; other confidentiality and data security issues | | 5 |
|  | **Data collection methods** - Types of data collected; details of data collection procedures including (as appropriate) start and stop dates of data collection and analysis, iterative process, triangulation of sources/methods, and modification of procedures in response to evolving study findings; rationale | | 5-6 |
|  | **Data collection instruments and technologies** - Description of instruments (e.g., interview guides, questionnaires) and devices (e.g., audio recorders) used for data collection; if/how the instrument(s) changed over the course of the study | | 5-6 |
|  | **Units of study** - Number and relevant characteristics of participants, documents, or events included in the study; level of participation (could be reported in results) | | 5-6 |
|  | **Data processing** - Methods for processing data prior to and during analysis, including transcription, data entry, data management and security, verification of data integrity, data coding, and anonymization/de-identification of excerpts | | 6 |
|  | **Data analysis** - Process by which inferences, themes, etc., were identified and developed, including the researchers involved in data analysis; usually references a specific paradigm or approach; rationale | | 6-7 |
|  | **Techniques to enhance trustworthiness** - Techniques to enhance trustworthiness and credibility of data analysis (e.g., member checking, audit trail, triangulation); rationale | | 6 |
|  |  | |  |
| **Results/findings** | | |  |
|  | **Synthesis and interpretation** - Main findings (e.g., interpretations, inferences, and themes); might include development of a theory or model, or integration with prior research or theory | | 8-14 |
|  | **Links to empirical data** - Evidence (e.g., quotes, field notes, text excerpts, photographs) to substantiate analytic findings | | 10-14 |
|  |  | |  |
| **Discussion** | | |  |
|  | **Integration with prior work, implications, transferability, and contribution(s) to the field -** Short summary of main findings; explanation of how findings and conclusions connect to, support, elaborate on, or challenge conclusions of earlier scholarship; discussion of scope of application/generalizability; identification of unique contribution(s) to scholarship in a discipline or field | | 15-19 |
|  | **Limitations** - Trustworthiness and limitations of findings | | 18 |
|  |  | |  |
| **Other** | | |  |
|  | **Conflicts of interest** - Potential sources of influence or perceived influence on study conduct and conclusions; how these were managed | | Title page |
|  | **Funding** - Sources of funding and other support; role of funders in data collection, interpretation, and reporting | | Title page |
|  |  | |  |
|  | |  |  |
|  | |  |  |
|  |  | |  |
|  |  | |  |
